# The assessment of antimalarial drug efficacy in-vivo

**DOI:** 10.1016/j.pt.2022.05.008

**Published:** 2022-06-06

**Authors:** Nicholas J. White

**Affiliations:** 1 Mahidol-Oxford Tropical Medicine Research Unit, Faculty of Tropical Medicine, Mahidol University, Bangkok Thailand; 2 Centre for Tropical Medicine and Global Health, Nuffield Department of Medicine, Oxford University, Oxford, UK

**Keywords:** malaria, antimalarial drugs, chemoprevention, pharmacometrics, treatment

## Abstract

Currently recommended methods of assessing uncomplicated falciparum malaria treatment work less well in high transmission than in low transmission settings. There is also uncertainty how to assess intermittent preventive therapies and seasonal malaria chemoprevention, and *P. vivax* radical cure. A “pharmacometric antimalarial resistance monitoring (PARM)” approach is proposed for slowly eliminated antimalarial drugs in areas of high transmission. In PARM antimalarial drug concentrations at recurrent parasitaemia are measured to identify outliers (i.e. recurrent parasitaemias in the presence of normally suppressive drug concentrations), and to characterise changes over time. PARM requires characterization of pharmacometric profiles but should be simpler and more sensitive than current methodologies. PARM does not require parasite genotyping, and can be applied to the assessment of both prevention and treatment.

## The changing focus of antimalarial drug assessment

Antimalarial drugs are used on a vast scale for both malaria prevention and treatment. Many of the drugs are slowly eliminated so the cumulative exposure in people living in malaria endemic areas is enormous. Resistance has now emerged to all widely available antimalarials, although the extent to which resistance affects therapeutic efficacy in prevention and treatment varies from minimal (e.g. *Pfmdr1* amplification for lumefantrine) to complete lack of effect (e.g. *Pfdhfr* I164L mutation conferring high level pyrimethamine resistance). Assessment of antimalarial therapeutic efficacy is needed to guide policies and practices. In the past, when use of failing drugs was widespread, it was important to focus on measuring efficacy in preventing or treating malaria. Now, that drugs are generally working well, and recrudescence rates are consequently very low, this approach is inefficient as it requires large studies to show small differences between treatments. In high transmission settings multiplicity of infection is usually high and reinfection is inevitable, and so it can be difficult to distinguish reinfection from recrudescence using multi allelic PCR genotyping approaches. Nevertheless, current methods of assessment focus on distinguishing the few recrudescences from the majority reinfections -which are not analysed further [1,2]. Yet these more numerous reinfections do provide valuable information on emerging resistance. They demonstrate the antimalarial drug concentrations which permit parasite growth in prevalent malaria parasites (levels which are below the infection’s minimum inhibitory concentration) [3,4]. Parasites which can grow in high drug concentrations are, by definition, resistant. Changes in antimalarial pharmacometrics in-vivo are more sensitive measures to assess developing resistance than current in-vivo tests. Their measurement from a single capillary blood sample in which malaria parasitaemia and antimalarial blood concentrations are quantitated provides a simple and readily applicable method of assessment which can identify early signs of emerging resistance to slowly eliminated drugs. This approach would allow preemptive changes in guidelines and thereby prevent or reduce the adverse impact of resistance on therapeutic efficacy [3]. This review assesses current methods of therapeutic assessment briefly [4] and proposes a new “simple pharmacometric evaluation (SPE)” approach for assessing the preventive and therapeutic efficacy of slowly eliminated antimalarials in high transmission settings

## Assessing malaria chemoprophylaxis

The objective of chemoprophylaxis is to prevent malaria (including asymptomatic infections in pregnant women). This can be achieved by preventing pre-erythrocytic development (causal prophylaxis e.g. primaquine, atovaquone) or by suppressing parasite multiplication in the blood stage infection only (suppressive prophylaxis e.g. chloroquine, mefloquine) [3]. Prophylactic efficacy and effectiveness are best assessed from randomised controlled, preferably double blind, comparative trials. The incidence of malaria is compared between the treatment arms and uses patent parasitaemia (assessed by microscopy) as the study’s primary end-point. If PCR is employed for detection or genotyping then high volume ultrasensitive PCR (which has a limit of detection around 20 parasites/mL [5]) should *not* be used if a suppressive prophylactic is being evaluated. This is because uPCR is so sensitive that it can detect the emergence of parasites from hepatic schizogony, which is not prevented by antimalarial drugs acting on blood stages only [6]. A breakthrough malaria infection during chemoprophylaxis may result from incorrect dosing, poor adherence, unusual pharmacokinetics (e.g. pharmacogenetics: “loss of function” CYP2D6 polymorphism contributing to reduced primaquine biotransformation), or drug resistant parasites. Adherence is a key determinant of chemoprophylactic effectiveness [7–9] so, where possible, chemoprophylaxis trials should include drug measurement. The timing and schedule of sampling depends on the drug’s elimination kinetics. For slowly eliminated drugs (terminal half-life >1 day), if possible, a blood level should be measured at the time of any breakthrough infection and malaria parasite DNA retained for resistance marker genotyping. Follow up should continue for at least one month after the subject leaves the endemic area.

It is widely stated that antimalarial chemoprophylaxis or chemoprevention provide strong selective pressure for the emergence of resistance. This generalisation is incorrect. For most drug resistance mechanisms effective preventive regimens provide very little selective pressure. This is because the only transmission opportunity is from infections emerging at the “tail” of the elimination profile after drug discontinuation [10].

## Assessing intermittent preventive therapies (IPT) and seasonal malaria chemoprevention (SMC)

Both these widely deployed interventions are forms of chemoprophylaxis recommended for use by apparently healthy people who live in malaria endemic areas. They are recommended for settings or periods where transmission is high (mainly sub-Saharan Africa and the island of New Guinea) [1,3,9,11]. The antimalarial drugs are typically given in treatment doses at monthly intervals or, in some cases, less frequently. SMC is given during periods of high transmission to children between 3 and 59 months of age, and IPT is given to infants with EPI immunisations (usually 2, 3, and 9 months) or during pregnancy (up to monthly in later pregnancy). The therapeutic objective is to clear any existing parasitaemia and to prevent malaria infection until the next dose is given or the indication has ceased [3,7–9]. Although these preventive therapies (see Box 1) are recommended by WHO [1] and extensively used, there is still not an accepted standardized approach to their assessment. This is particularly important for sulfadoxine-pyrimethamine (SP), the main drug used for IPT, which has fallen to resistance in many areas. As IPT and SMC are recommended in higher transmission settings, they are assisted by significant immunity in the first months of life (from maternal antibodies), in older children and in adults (e.g. IPT in pregnant women [9,11]). Unless drug resistance levels are very high, preventive efficacy is always superior to treatment efficacy. This is because the parasite numbers in the blood after hepatic schizogony are at least three orders of magnitude lower than in acute malaria, and the parasiticidal effect required to prevent multiplication is over two orders of magnitude lower than required to clear an established infection [3,4,10]. As a consequence, drugs which are insufficiently efficacious in treatment because of resistance (e.g. SP) may still be useful in prevention. However, as resistance worsens, eventually preventive efficacy is lost too.

**Figure 1:**
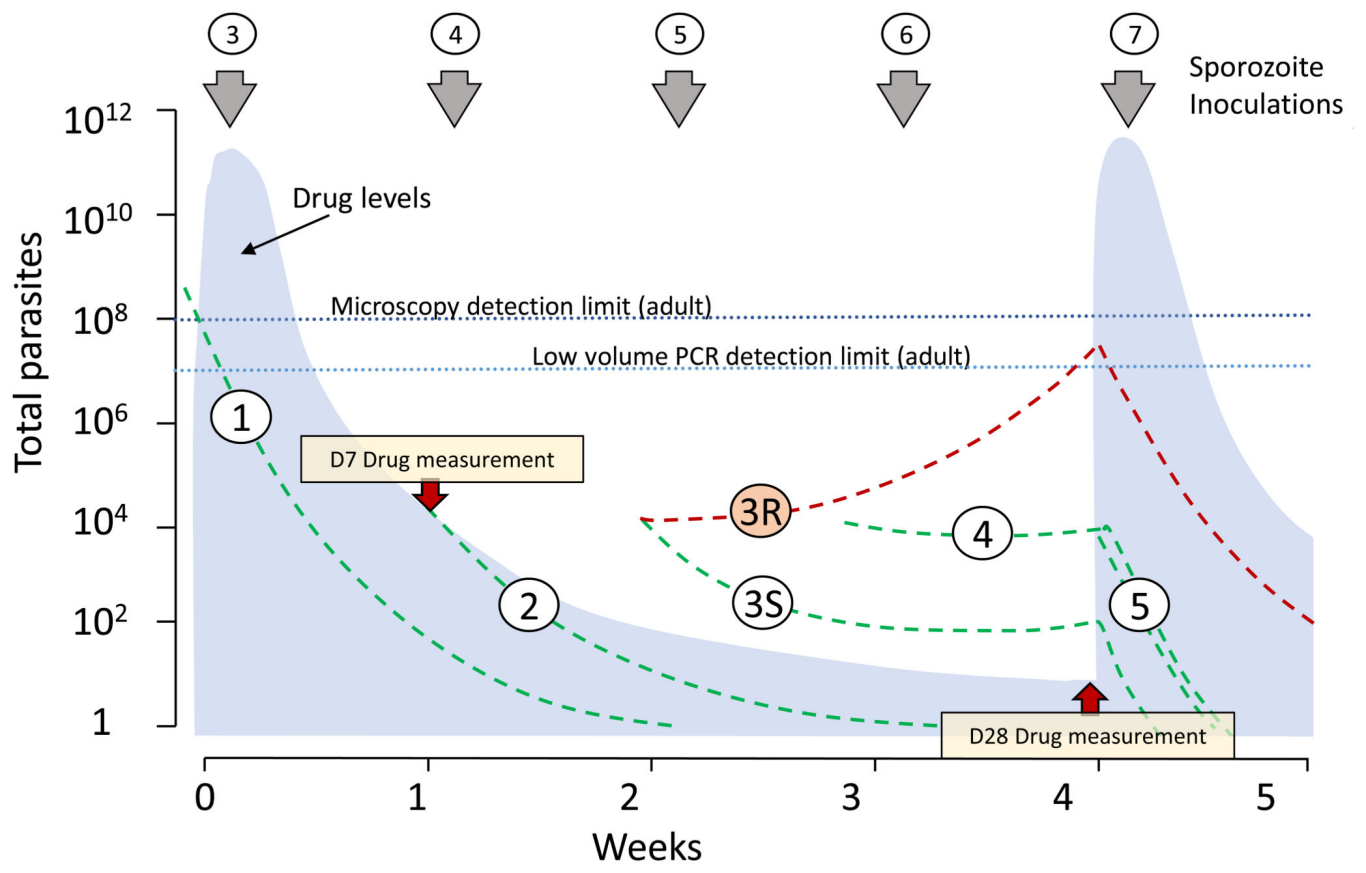
Preventive antimalarial pharmacometrics in a high transmission setting. The total number of parasites in the body (y axis log scale) of a parasitaemic pregnant woman who starts IPTp are shown as dashed lines [9,11]. She is bitten by sporozoite bearing anopheline mosquitos on average once each week (EIR 50/year). Her second next antenatal clinic visit and second IPTp dose is one month later. The incubation period is 13 days and approximately 30,000 parasites are liberated at hepatic schizogony. The blood concentration profile of the IPT drug is shown in light blue. Drug exposure in the population can be characterized by measuring day 7 concentrations [27]. Sensitive parasites (green dashed lines) are either cleared (infection 1 was patent but asymptomatic, and was present before starting IPT, infection 2 was cleared after emerging from the liver) or they are suppressed (infections 3-5) until the next round of IPT which then clears them. A resistant infection (3R) acquired when IPT started, emerges from the liver 13 days later and soon begins to expand reaching densities which are detectable by PCR, but not by microscopy, before the next round of IPT. Drug levels measured at the time reveal antimalarial drug levels which should have suppressed parasite growth (as in 3S) thereby distinguishing inadequate exposure from drug resistance as a cause of recurrent infection. In practice many women have longer intervals than one month between doses.

In a high transmission setting reinfection is inevitable. Antimalarial drugs which are slowly eliminated delay the appearance of reinfection until the drug levels have fallen below the prevalent minimum inhibitory concentrations (MICs) [4,12–14] (Figure 1). Effective IPT or SMC either eliminates the reinfections or prevents them reaching patent densities which may cause illness before the next treatment dose is given. Reinfections in older children receiving SMC and pregnant women receiving IPTp are usually asymptomatic so passive detection of illness is insensitive as a measure of efficacy. Furthermore, the adverse effects of malaria in pregnancy on intrauterine growth, and thus birthweight, occur even though the mother has no symptoms [9, 11]. Sensitive methods are therefore needed for therapeutic assessment of these preventive therapies.

Efficacy can be assessed simply by screening for parasitaemia immediately before the next treatment dose (Figure 1). Screening could be either by microscopy (LOQ ˜ 50,000 parasites/mL) or by blood spot PCR (LOQ 1000-5000 /mL) (but not by high volume PCR (LOQ 22/mL) for the reasons explained above). The reinfection risk is often seasonal, so the assessment has greatest statistical power at the time of peak transmission intensity. As the regrowth of parasites is slowed by partially suppressive antimalarial blood concentrations, a finger prick blood sample PCR (limit of detection 1-5 parasites/μL) provides greater diagnostic sensitivity than a blood smear or rapid diagnostic test (Figure 1). But it is still important to quantitate the parasite density, either by qPCR or blood smear. The probability of detecting recurrent parasitaemia depends on the frequency of inoculation (EIR), the timing of reinfection (likely random), the antimalarial drug concentration profile (which determines the asexual parasite multiplication rate), and the levels of parasite drug susceptibility and host immunity [4]. To interpret the results, a concomitant blood concentration of the antimalarial drug should be measured also [13]. There are validated filter paper methods for measurement of several of the antimalarial drugs in dry blood spots which facilitate field assessment [15]. Levels of pyrimethamine (t_½β_ ~ 4 days) may not be detectable 28 days (7 half-lives) after drug administration, but the other widely used preventive medicines (sulfadoxine, piperaquine, desethylamodiaquine) should be measurable with currently available sensitive detection assays. Population pharmacokinetic evaluations of these drugs in different populations have been performed to calibrate the expected range of concentrations (16-22) (although more studies are needed). The exact design of a surveillance study will depend on the elimination kinetics of the drug, the sensitivity of the drug assay and the drug administration schedule. Thus, a single finger -prick blood sample taken approximately one month after drug administration (i.e. often immediately before a subsequent dose) provides all the necessary information. Finding malaria parasites together with very low or undetectable drug levels suggest either poor adherence or unusual pharmacokinetics, whereas expected drug levels suggest reduced parasite drug susceptibility (Figure 1). If there are validated molecular markers of antimalarial drug resistance, these can be evaluated in the same sample if there is sufficient DNA. Samples from recurrences in the presence of relatively high drug concentrations are also valuable for genome wide association studies investigating molecular correlates of resistance. In a high transmission setting it is expected that the first reinfections to establish after administering a slowly eliminated antimalarial will be with more resistant parasites. From a clinical standpoint, the question is whether these are occurring within the chemoprevention dosing interval. From an epidemiological standpoint the question is whether there is a trend towards earlier reinfections (i.e. are infections becoming detectable at higher blood concentrations suggesting emerging resistance?). Time to patent reinfection alone would also be a useful indicator of drug susceptibility, but this requires active follow up with frequent blood sampling, in order to identify asymptomatic reinfections. Taking a single sample before the next dose of IPT or SMC (i.e. about 4 weeks after drug administration) and assessing the proportion with detectable parasitaemia is simpler and more acceptable. Even if all samples are parasite negative, the result is informative. For example, if 100 subjects receiving IPT or SMC were tested and none had detectable parasitaemia at 28 days, then the upper 95% confidence interval for the proportion of breakthrough infections is 1 in 33. This simple approach to the assessment of preventive efficacy would be much easier, more accurate, and more sensitive than the application of TES methodology designed to assess treatment efficacy (i.e. blood microscopy at days 2, 7, 14, 21 and 28 with genotyping of paired isolates and without drug measurement), which is currently under consideration by WHO [2]. Asymptomatic infections which recur within two weeks of a treatment dose must be highly resistant and should have precluded consideration of the preventive therapy.

The adverse consequences of malaria in pregnancy in higher transmission settings result from the duration and extent of malaria infection and placental sequestration [9, 11]. This can be assessed both by screening for parasitaemia during the pregnancy, and also by assessing placental parasitisation at delivery in relation to birthweight.

## Assessing the treatment of uncomplicated malaria

**Figure 2:**
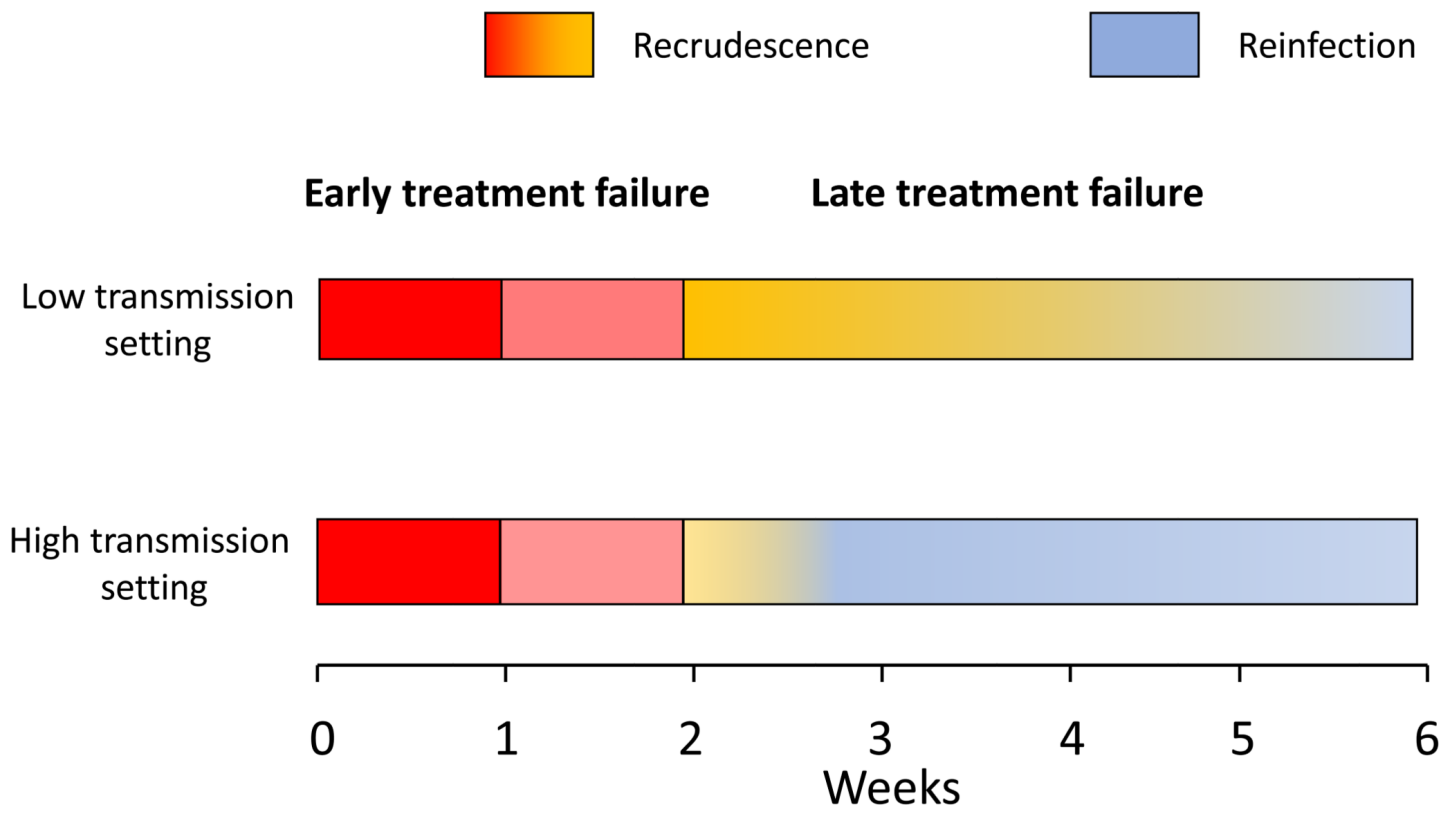
Assessing treatment responses in symptomatic malaria. An increase in severity and malaria parasitaemia or a failure to clear parasitaemia within 7 days constitutes an early treatment failure (red) which results either from failure to take or absorb the antimalarial drug, or high-grade resistance. From 7-14 days reinfection is not possible unless there is a very high level of resistance [2–4] Nearly all recurrences in the second week are recrudescences. After 14 days patent reinfection is possible, but this still implies low drug exposure or resistance. In low transmission settings recrudescence is more likely. If drug exposure is adequate this implies a lower level of resistance (yellow) than for earlier treatment failures, whereas in high transmission settings reinfection is more likely, and may preempt recrudescence. Recurrences, whether recrudescences or reinfections, have to grow through the declining concentrations of the slowly eliminated antimalarials. The first reinfections to establish are the most resistant [4].

Methods of assessing oral antimalarial drugs in uncomplicated falciparum malaria have evolved erratically over the past 50 years. Follow-up periods to identify recurrences were initially 28 days, then 14 days and, over the past two decades, have been extended to 4-9 weeks depending on the elimination kinetics of the tested drug [2–4, 23] (Figure 2). Over this period antimalarial treatments have improved substantially. Higher levels of efficacy and effectiveness are now required of antimalarial treatment regimens. Whereas 75% efficacy assessed at two weeks was once considered acceptable by some, now ≥95% efficacy assessed at ≥28 days is the therapeutic target. Policy change is recommended if efficacy falls below 90% [1,3]. With current treatments the emphasis has therefore moved from quantifying treatment failure rates to early detection of resistance. The first-generation studies kept the volunteer patients away from reinfection (in cities where there was no transmission or in closed mosquito proofed wards) [24]. The initial community-based studies conducted in endemic areas, where reinfection cannot be excluded, adjusted for reinfection rates from concomitant epidemiological information. This adjustment was improved and simplified by the introduction of parasite genotyping to distinguish recrudescence from reinfection. This PCR “genotyping correction” uses polymorphic genetic markers to assign probabilities that a recurrent malaria infection is the same or different to that which caused the original infection [25]. This requires sufficient genetic diversity both in the parasite population, and in the markers used, such that the probability of reinfection with an identical genotype is very low. The most commonly used PCR genotyping approach involves comparison of polymorphic segments of the MSP1, MSP2 and GLURP genes [25, 26]. Despite many theoretical objections, this approach has resulted in a substantial increase in the number of therapeutic assessments and clinical trials, thereby providing very valuable information over the past 25 years. In a standard therapeutic assessment, the full course of quality assured antimalarial, at recommended doses, is administered under observation [2]. Weight and height should be recorded, and patients should be observed for at least one hour in case of vomiting. The patient should be followed daily until fever and parasite clearance with a minimum of once daily parasite counts.

### Alternatives to standard in-vivo assessments which focus on recrudescence

**Figure 3:**
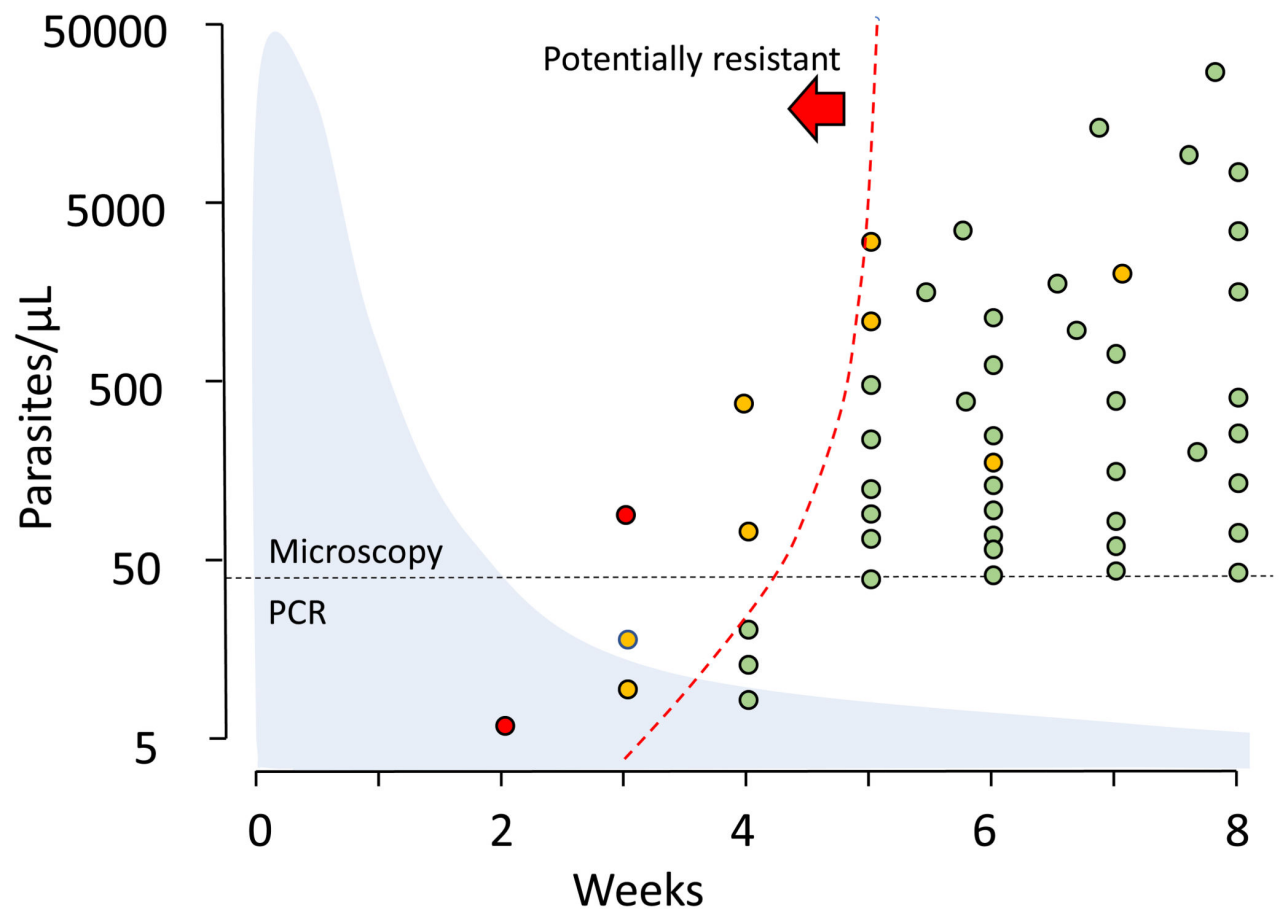
Detecting antimalarial resistance in vivo. Example of the pattern of *P. falciparum* reinfections in an area of high transmission following an ACT for uncomplicated malaria. Weekly follow up for 8 weeks. Finger prick blood samples for PCR were taken in weeks 2, 3, and 4. Blood slides were assessed weekly {4]. Microscopy can detect parasite densities down to around 50/μL, and finger prick capillary blood sample PCR down to 1-5/ μL. As the blood concentrations of the slowly eliminated partner drug decline (light blue shadow shows average drug concentration profile), infections are progressively able to establish and multiply. Some patients present with symptomatic recurrences between the follow-up days. The dashed line shows a boundary to the left of which detectable recurrent parasitaemias may be resistant. This requires definition. It could be defined as 99% of drug sensitive parasites could not reach detectable parasitaemias if exposed to the geometric mean antimalarial drug plasma concentration. Recurrent parasite densities appropriate for sensitive *P. falciparum* are shown in green. The individual contemporaneously measured drug concentration, and thus the preceding drug exposures, were insufficient to suppress the growth of sensitive parasites. Recurrences associated with low drug concentrations are shown in yellow. Recurrences which are outside the 99 percentile for sensitive *P. falciparum* for the individual contemporaneously measured drug concentration are shown in red. These parasites have grown in concentrations of antimalarial drug which should have suppressed the growth of sensitive parasites. They are therefore likely to be drug resistant [13, 14]. Pharmacometric studies are required to calibrate these thresholds.

Therapeutic assessments should be conducted in both adults and children in low transmission settings whereas in higher transmission settings, children only should be studied. Enrolling non-pregnant adults with asymptomatic parasitaemia in antimalarial drug studies is misleading as they will self-cure readily, and the benefit of the antimalarial drugs will be overestimated. In high transmission areas people are infected frequently, commonly harbour several different parasite lineages, and rapidly become reinfected as antimalarial drug concentrations fall below MIC values (Figure 3). This complicates the interpretation of current PCR genotyping methods relying on 1-3 polymorphic loci. The difficulty in interpreting genotyping results has prompted calls for research to evaluate more detailed (i.e. more loci, longer reads) genotyping methods and apply these in standard WHO in-vivo monitoring tests [26].

**Figure 4:**
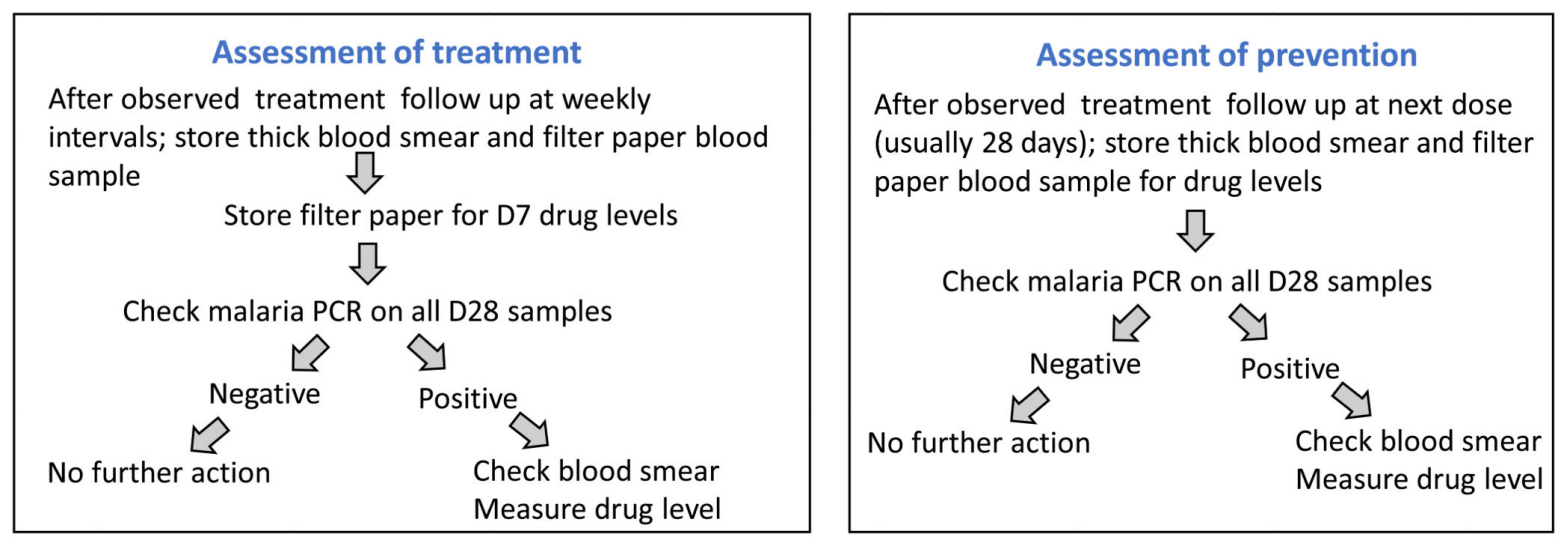
A suggested algorithm for simple pharmacometric evaluation. D7 levels can be added to the prevention assessment, in which case the only difference with treatment assessment is that weekly follow up is not essential.

An alternative approach to the evaluation of slowly eliminated antimalarials in high transmission settings is simply to measure the drug concentrations at the time of parasitaemia recurrence, and to ignore the differentiation of reinfection or recrudescence initially (Table 1). Antimalarial treatments with slowly eliminated antimalarials (as in most ACTs) should prevent reinfection becoming detectable within one month so a symptomatic recurrence of malaria within 28 days of treatment is a “treatment failure”, whether or not it is a recrudescence [2]. Measuring the drug concentration at the time of recurrence (or earlier in all study patients at day 7 [27]) distinguishes inadequate exposure from drug resistance. This could be simplified operationally to a single evaluation in all study patients at 28 days. If the prevalence of parasitaemia at 28 days is high (e.g. > 5%) then earlier evaluation (e.g. D21) can be added. As described above, recurrences have to grow through the declining concentrations of the slowly eliminated antimalarials. This heterogeneous concentration gradient acts as a filter. If resistance increases steadily, or in modest increments (e.g. stepwise acquisition of *Pfdhfr* mutations), then the first reinfections to establish are usually the most resistant [4] (Figure 3). In high transmission settings increasing resistance will result in earlier reinfections, so testing the drug concentrations, and molecular markers (if known), in blood samples at the time of detected recurrence provides a sensitive early warning of antimalarial drug resistance -well before overt treatment failures occur. This is because early recurrence precedes recrudescence as resistance worsens [4]. Valuable information is therefore gained even in trials in which there are no treatment failures. There are already sufficient profiles of concentration data, either measured or modelled, at detection of reinfection for some drugs, but more information is needed to define the probability distributions and set threshold criteria for outliers. A simple, practical, potentially high throughput approach is shown in Figure 4.

## Assessing rapidly eliminated drugs

PARM is not suitable for evaluating rapidly eliminated antimalarial drugs. If artemisinin resistance is suspected, or a ring stage drug action is being evaluated, then parasite counts should be measured at least three times each day until levels become undetectable [28, 29]. Ring stage activity is assessed from the slope or half-life of the initial log-linear decline in circulating parasite densities. Immunity accelerates parasite clearance, so clearance rates are faster in higher transmission settings [30]. Assessing the parasite clearance slopes accurately requires sufficiently high initial parasite densities (>10,000/μL) and, preferably, staging of parasite development before treatment [31]. Parasite clearance rates are best expressed as half-lives (PC_1/2_) [29]. In low transmission settings PC_1/2_ values over 5 hours point towards artemisinin resistance (usually associated with point mutations in the propeller region of the *Pf*kelch gene) although some patients with wild-type infections may still clear their parasitaemia slowly (PC_1/2_ values >5 hours [30, 32]. Following treatment with artemisinin derivatives, low densities (detectable by PCR) of apparently dormant persisting parasites may be observed for more than a week after starting treatment [33].

## Assessing radical curative efficacy

*P. vivax* and *P. ovale* infections can relapse weeks or months after the primary infection. The relapses arise from activation of dormant liver stages “hypnozoites”. The only drugs which unequivocally prevent relapse (known as radical cure) are the 8-aminoquinolines (primaquine, tafenoquine). There is no generally agreed methodology for assessing radical cure [34–36]. In endemic areas relapse cannot be distinguished reliably from recrudescence or reinfection as the relapses can be with similar or with different genotypes to those which caused the initial infection. Combining genotyping with time-to-event modelling improves probabilistic discrimination of relapse from reinfection [35]. Most *P. vivax* now is of the tropical frequent relapse type [34] so a six month follow up will capture most relapses [37, 38]. However, if long latency *P. vivax* is present (e.g Koreas, Northern India) then one year’s follow is required as these strains relapse 8-9 months after the primary infection [34, 36, 38]. In assessing drug resistance, the principles are the same as for *P. falciparum.* Following artesunate or quinine treatments, both of which are eliminated rapidly, the incidence of relapses typically peaks around three weeks after starting treatment [35]. Slowly eliminated drugs delay relapses of tropical *P. vivax* [13, 14]. Following artemether-lumefantrine many *P. vivax* relapses appear after four weeks, and after chloroquine, pyronaridine, mefloquine, or piperaquine they usually appear after six weeks. As for reinfections of *P. falciparum* in high transmission settings, the first sign of drug resistance in *P. vivax* infections is earlier relapses [13, 14]. Measuring the blood concentrations of antimalarial on day 7 [27], and at the time of recurrence allows distinction between resistance and inadequate drug exposure. Finding *P. vivax* parasitaemia in the presence of blood chloroquine concentration of >100ng/mL has been regarded as evidence of resistance in *P. vivax* [39].

## Assessing transmission blocking activity

Only *P. falciparum* has mature gametocytes which are insensitive to the treatment antimalarial drugs [40], and therefore require separate assessment. A minority of symptomatic malaria patients have patent *P. falciparum* gametocytaemia at presentation, whereas slowly cleared gametocytes comprise a major proportion of asymptomatic parasitaemias. Gametocytocidal activity is conventionally assessed by comparing gametocyte carriage (duration or area under the curve) [41]. This can be used to compare potentially transmission blocking drugs, but it is insensitive and potentially inaccurate as gamete sterilization occurs much more rapidly than clearance [41]. The best way to compare drugs or doses is to assess infectivity to anopheline mosquitoes and compare oocyst and later sporozoite formation [40–42]. However, this is difficult as it requires substantial entomology support and expertise, and it is consequently seldom performed.

## Assessing the treatment of severe malaria

**Figure 5:**
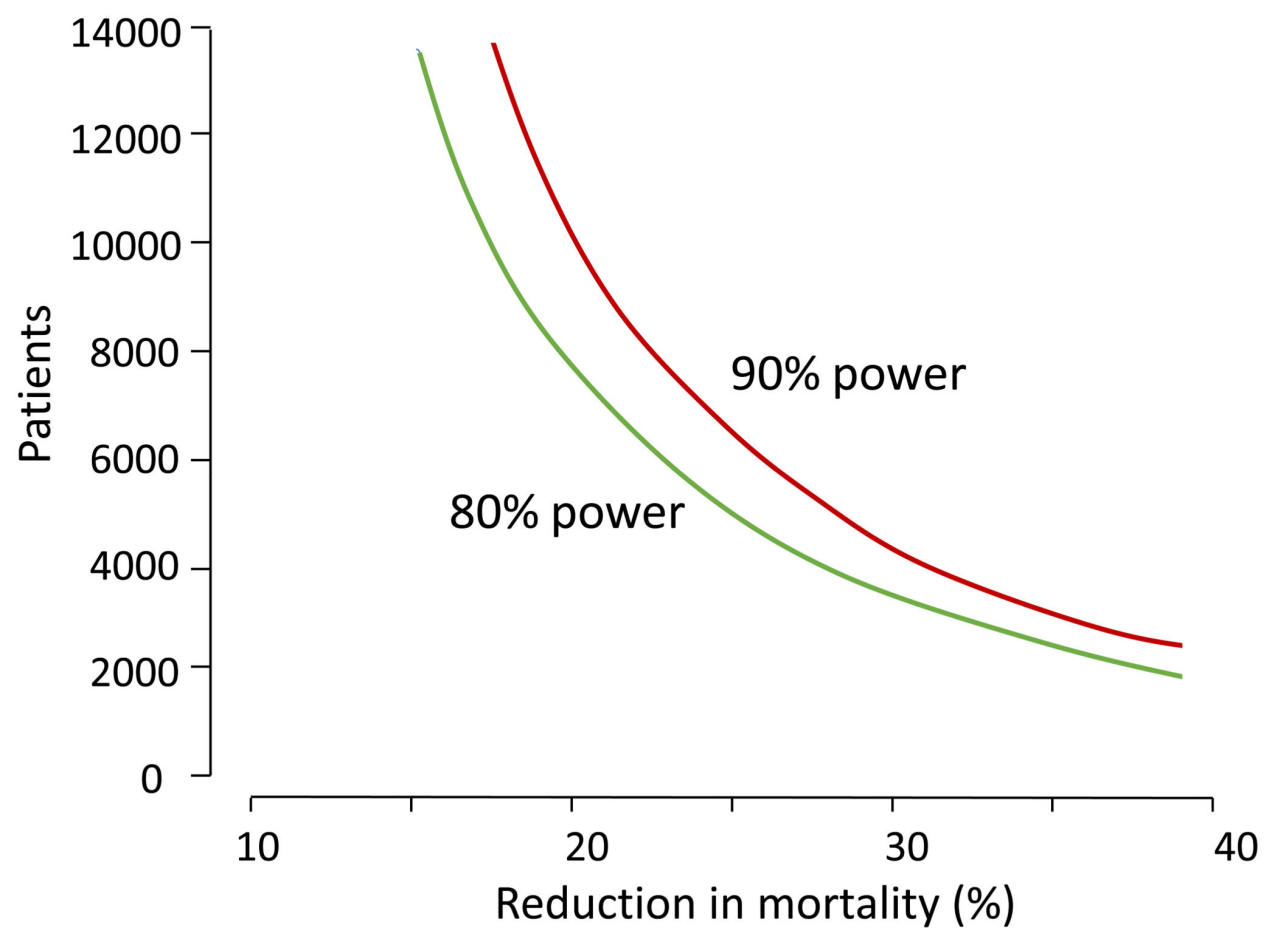
Severe malaria trial sample sizes. Total numbers of patients in relation to the reduction in mortality required in a superiority randomized controlled trial (with 95% confidence) in childhood severe malaria in which the comparator was artesunate (mortality 8.5%) with 1:1 randomisation [43].

The primary objective of severe malaria treatment is to save life [43]. To demonstrate differences in mortality very large randomized controlled trials are needed. For example, the AQUAMAT trial, which led to the replacement of quinine by artesunate in Africa, enrolled 5425 children with severe falciparum malaria [44]. The mortality in the quinine group (previous standard of care) was 11.0% compared with 8.5% in the artesunate treated children; a relative reduction of 22.5% (95% CI 8.1 to 36.9%, p=0.002). Demonstrating a further 20% reduction in mortality in comparison to artesunate (which has now become standard of care), in an RCT with 95% confidence and 80% power and 1:1 randomisation, would require a trial of 7676 patients (Figure 5). Several randomized trials and observational studies in “severe malaria” have had more generous criteria for inclusion and much lower mortalities as consequence. These would require even larger sample sizes to demonstrate mortality differences. It is also increasingly clear that the diagnosis of severe malaria in African children is often wrong. It has been estimated recently that about one third of African children diagnosed as having severe malaria have incidental parasitaemia or uncomplicated malaria, and another cause of severe illness (often sepsis) [45]. As this misdiagnosed subgroup has a higher mortality than “true severe malaria,” it dilutes substantially the power of randomized controlled trials assessing malaria specific interventions. Use of biomarkers such as plasma *Pf*HRP2 levels (high), platelet counts (low), plasma *P. falciparum* DNA (high), pigment containing neutrophils on blood films (specific) and, less informatively, neutrophil counts substantially increases the specificity of the severe malaria diagnosis in African children and should always be evaluated in clinical trials [45, 46]. In low transmission settings, where the majority of severely ill patients are adults, and incidental microscopy-detectable parasitaemia is unusual, the diagnosis is much more specific [43].

As conducting large and definitive trials with mortality as the primary end point is difficult there has been discussion over the validity of surrogate measures which reflect resolution of the disease process [47]. These could be used to prioritise interventions for larger scale evaluation. Clinical recovery rates are informative-particularly times to recover from coma and rate of resolution of metabolic acidosis or hyperlactataemia [47]. However, there is a competing risk in that a drug which improves survival will save some of the most seriously ill patients who might die with the other treatment. As the time to recovery depends on the severity of illness, these survivors will have the slowest clinical recovery times. This biases the measure against the more effective treatment. In the severe malaria trials evaluating artemisinin derivatives coma recovery times in cerebral malaria were often longer in the artemisinin treated group for this reason. Resolution of hyperlactataemia or metabolic acidosis may be the best surrogate measure [43]. In our studies we have measured venous blood or plasma lactate four hourly for the first 12 hours, and then six hourly. Other indices which may be compared are rates of recovery from renal impairment, degree of anaemia, or incidence of complications such as seizures

## Covariates

Malaria is a heterogeneous disease ranging in severity from oligosymptomatic to rapidly lethal [43]. Rates of clinical and parasitological recovery should be adjusted for baseline values. In therapeutic assessments symptomatic patients with very low parasite densities may be excluded, either because in a high transmission settings it may be difficult to ascribe the infection to malaria, or because parasite clearance rates are being assessed primarily, and these cannot be assessed reliably if admission parasite counts are low [28, 29]. Patients with very high parasitaemias should be excluded also (exact thresholds are debated – working primarily in low transmission settings we use a threshold of 4% parasitaemia for “uncomplicated hyperparasitaemia”) because these patients have a much higher risk of progressing to severe malaria, and they have a substantially higher risk of treatment failure (approximately six fold on the Western border of Thailand) [48, 49]. For this reason, and because they are the potential source of de-novo resistance [50], they are a very important but generally neglected patient sub-group which requires specific study.

As immunity is such a powerful contributor to protection from malaria, and an accelerator of recovery, therapeutic responses must be interpreted against the likely background immunity [51]. Preventive therapies will be more effective in partially immune individuals. For example, in some areas sulfadoxine-pyrimethamine remains effective in preventing the adverse consequences of malaria in pregnancy (primarily low birthweight), while it is relatively ineffective as a treatment of malaria in children. In the treatment of malaria therapeutic response should be stratified by age. In low transmission areas this is usually divided as 0-5, 6-15 and >15 Years.

## Concluding remarks

Pharmacometric antimalarial resistance monitoring of therapeutic efficacy allows earlier identification of resistance to slowly eliminated antimalarial drugs than conventional therapeutic efficacy studies. PARM is a suitable approach for the evaluation of both antimalarial prevention and treatment in areas of high malaria transmission. It places greater emphasis on drug measurement than on parasite genotyping. It is a new paradigm that would need pharmacometric studies to optimize design for each evaluated antimalarial and to set thresholds. There are a number of important outstanding questions which need addressing (see box). If PARM was adopted, it would require investment in antimalarial drug measurement in Africa.

## Figures and Tables

**Table 1:** Assessing antimalarial efficacy in high transmission settings.

	Focus	Advantages	Disadvantages
Conventional (TES) in-vivo testing	Distinguishing recrudescence from reinfection and measuring recrudescence rate	Provides an estimate of the treatment failure rate Does not require calibration	Insensitive measure of emerging resistance Genotyping errors compromise estimates Requires large sample sizes for accurate estimates Confounded by pre-treatment Does not distinguish resistance from inadequate exposure Not applicable to asymptomatic malaria or preventive approaches (SMC, IPT)
Alternative simple pharmacometric evaluation (PARM)	Characterising the antimalarial blood concentrations at which recurrences become detectable	Simple Much greater statistical power (as reinfections occur eventually in all subjects) Does not require genotyping Not confounded by pre-treatment Provides early warning of emerging resistance. Distinguishes resistance from inadequate exposure Applicable to all prevention and treatment evaluations	Requires calibration for each drug Requires drug measurement Does not distinguish recrudescence from reinfection (although genotyping could be added)

## Box 1 Preventive therapies

**Chemoprophylaxis:** Continuous administration of antimalarial drugs either daily or weekly to prevent malaria

**Intermittent Preventive Therapy** (IPT): Administration of full treatment doses at intervals of one month or more (to pregnant women: IPTp), or with EPI vaccinations at 2,3 and 9 months to infants (IPTi) to prevent malaria. Sulfadoxine-pyrimethamine (SP) is the principal drug used despite increasing resistance.

**Seasonal Malaria Chemoprevention** (SMC): Administration of full treatment doses at monthly intervals to all children between 3 and 59 months during the period of highest malaria transmission to prevent malaria. Amodiaquine +SP is the usual SMC. It has been given to 25 million children across the Sahel region each year during the 3-4 month rainy season.
